# The long-term effects of an implantable drop foot stimulator on gait in hemiparetic patients

**DOI:** 10.1371/journal.pone.0214991

**Published:** 2019-04-17

**Authors:** Agnes Sturma, Othmar Schuhfried, Timothy Hasenoehrl, Clemens Ambrozy, Stefan Salminger, Laura A. Hruby, Johannes A. Mayer, Kirsten Götz-Neumann, Richard Crevenna, Michaela M. Pinter, Oskar C. Aszmann

**Affiliations:** 1 Christian Doppler Laboratory for Restoration of Extremity Function, Medical University of Vienna, Vienna, Austria; 2 Master’s Degree Program Health Assisting Engineering, University of Applied Sciences FH Campus Wien, Vienna, Austria; 3 Department of Bioengineering, Imperial College London, London, United Kingdom; 4 Department of Physical Medicine, Rehabilitation and Occupational Medicine, Medical University of Vienna, Vienna, Austria; 5 Division of Plastic and Reconstructive Surgery, Department of Surgery, Medical University of Vienna, Vienna, Austria; 6 Department of Orthopaedics and Trauma Surgery, Medical University of Vienna, Vienna, Austria; 7 Observational Gait Instructor Group (O.G.I.G.), Los Angeles, United States of America; 8 Department for Clinical Neurosciences and Preventive Medicine, Danube-University Krems, Krems, Austria; 9 Neurological Rehabilitation Center Allentsteig, Allentsteig, Austria; Shanghai Jiao Tong University, CHINA

## Abstract

Drop foot is a frequent abnormality in gait after central nervous system lesions. Different treatment strategies are available to functionally restore dorsal extension during swing phase in gait. Orthoses as well as surface and implantable devices for electrical stimulation of the peroneal nerve may be used in patients who do not regain good dorsal extension. While several studies investigated the effects of implanted systems on walking speed and gait endurance, only a few studies have focussed on the system’s impact on kinematics and long-term outcomes. Therefore, our aim was to further investigate the effects of the implanted system ActiGait on gait kinematics and spatiotemporal parameters for the first time with a 1-year follow-up period. 10 patients were implanted with an ActiGait stimulator, with 8 patients completing baseline and follow-up assessments. Assessments included a 10-m walking test, video-based gait analysis and a Visual Analogue Scale (VAS) for health status. At baseline, gait analysis was performed without any assistive device as well as with surface electrical stimulation. At follow-up patients walked with the ActiGait system switched off and on. The maximum dorsal extension of the ankle at initial contact increased significantly between baseline without stimulation and follow-up with ActiGait (p = 0.018). While the spatio-temporal parameters did not seem to change much with the use of ActiGait in convenient walking speed, patients did walk faster when using surface stimulation or ActiGait compared to no stimulation at the 10-m walking test at their fastest possible walking speed. Patients rated their health better at the 1-year follow-up. In summary, a global improvement in gait kinematics compared to no stimulation was observed and the long-term safety of the device could be confirmed.

## Introduction

In neurologic conditions such as stroke, multiple sclerosis or traumatic brain injury a functional impairment in gait is often seen. One frequent abnormality is the inability to extend the ankle during swing phase. This so-called “drop-foot” has an estimated incidence of 20% after stroke [1]. In normal gait, dorsal extension of the foot is needed in the swing phase to ensure adequate foot clearance [2–4]. When hemiparetic patients are not able to actively extend their ankle, they compensate to gain sufficient clearance for the leg to swing [5]. These compensatory mechanisms vary between patients and may include increased plantarflexion of the less/non-affected side, hip abduction and pelvic tilt of the affected side or lateral flexion of the trunk [6, 7]. As compensation is often insufficient or inefficient, patients tend to drag the hemiparetic foot across the ground with a high risk of falls [8, 9]. Additionally, the abnormal gait pattern leads to slower walking speed and, together with the increased risk of fall [10], they cause a reduced feeling of safety, necessitating the use of walking aids [11]. Compensatory mechanisms may also cause additional physical defects, including overuse symptoms due to increased activity in other muscle groups (such as tibialis posterior, flexor hallucis and digitorum, semitendinosus and semimembranosus) [12]. The force integral for these muscles has been found to increase by more than 200% in a drop-foot group as compared to a control group [12, 13]. Additionally, other compensatory movements along the mechanical axis including circumduction, pelvis obliquity and hip flexion [13, 14], have been associated with increased mechanical energetic cost during walking [7]. However, in the absence of other neurophysiological improvements, compensation strategies need to be interpreted as a valuable alternative to improve functional mobility [15].

Restoration of normal gait function is a main goal in the rehabilitation of patients with a hemiparesis [10]. As gait impairments lead to difficulties in performing activities of daily living, their restoration is expected to translate into increased participation in daily life activities and therefore improve overall quality of life [10, 16].

Assistive devices to improve gait function with drop foot include orthoses as well as surface and implantable devices for electrical stimulation [17]. Orthoses and electrical stimulation of the peroneal nerve can both be considered safe in their (long-term) use in chronic stroke patients and were reported to facilitate clinically important changes in gait speed [18] and activity level [19]. Previous studies have also outlined the benefits of surface electrical stimulation on gait and quality of life in patients with drop foot [20, 21]. Electrical stimulation has been shown to be superior to ankle foot orthoses regarding knee stability, ankle dorsal extension power, propulsion [22] the ability to negotiate a sudden obstacle [23] and based on patients’ preferences [19, 24]. However, literature is inconclusive on whether functional electrical stimulation should always be preferred over ankle foot orthoses [15] and both seem to be equally effective regarding increases in walking speed and activity level [19, 24].

While surface electrical stimulation can cause chronic skin problems [25], implanted systems for functional electrical nerve stimulation bypass this disadvantage and, more importantly, circumvent the problem of correct electrode placement [26].

During the past decade a few studies have investigated the effects of implanted systems on gait [27–32] and quality of life [26, 30, 32]. Most of them found an increase in walking speed and dorsal extension of the ankle during swing phase and/or initial contact as well as good patient-reported device satisfaction and improved quality of life. These studies, however, had relatively short follow-up periods of a few weeks to half a year. While the positive effects of implantable systems described in these studies are promising, longer follow-up periods are needed to evaluate long-term effects on gait considering that the devices are designed to be used for decades. We hypothesized that time plays an important role in terms of the system’s benefits on a patient’s gait, as patients might need an extended period of time to get used to the drop foot stimulator. While the system is usually activated four to five weeks after implantation, usage is restricted to a few hours a day for up to another six weeks. Consequently, gait parameters were analysed with a 1-year-follow-up to uncover long-term effects, which we hypothesized to further change with increased usage time. In this article, we therefore present the first investigation with a 1-year follow-up describing the benefits of the implantable drop-foot stimulator ActiGait (Ottobock Health Care, Duderstadt, Germany). Our aims were to investigate the effect of the device on kinematic and spatio-temporal gait parameters as well as present patient-reported data to bridge the gap between objective outcome measures and a patient’s subjective well-being. Considering that implanted electrical stimulation has been reported as an option for patients who have used surface stimulation, but get skin problems [25] we also aimed to include surface stimulation in the comparison.

## Methods

### Ethical statement

The data presented here were collected in a clinical setting to document outcomes of the procedure. This study was approved by the Institutional Review Board of the Medical University of Vienna, Austria. The approval number was 2126/2016. Additionally, this study was registered at ClinicalTrials.gov with the registration number NCT03447717. As it presents retrospective analysis of anonymous data obtained in clinical work, no informed consent was given. All tests were conducted by the same team of experienced clinical researchers and none of the researchers reported a conflict of interest.

### Participants

**Fig 1:**
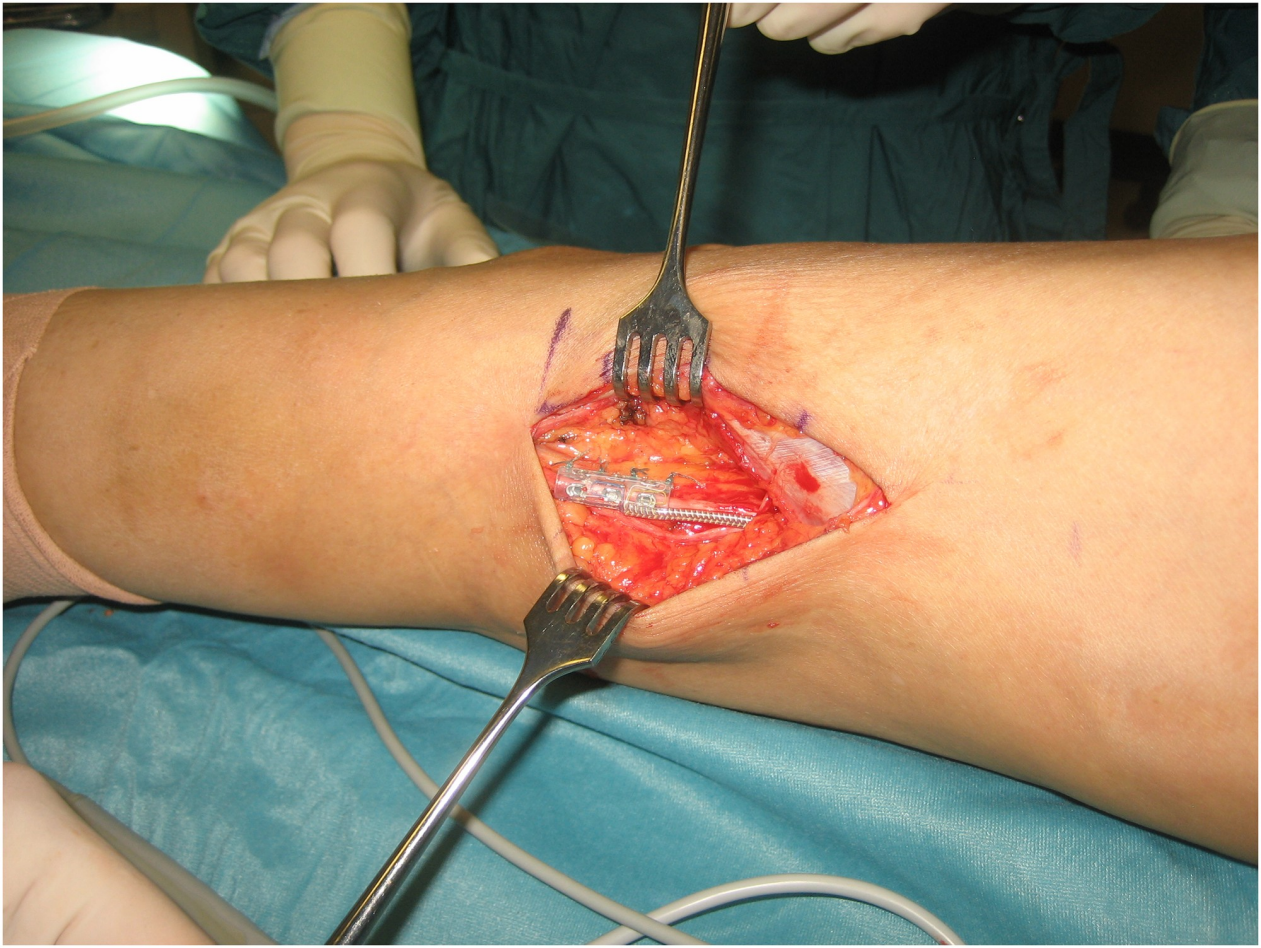
Flowchart displaying the progress of all participants through clinical testing and data analysis.

Patients with a chronic neurologic condition and a drop-foot were offered the possibility to have the ActiGait device implanted. They were recruited from our institutions as well as from a rehabilitation centre for neurologic conditions and were initially seen between 2011 and 2015. Inclusion criteria were a drop foot after stroke (defined as the inability to perform dorsal extension against gravity, which is equivalent to a British Medical Research Council (BMRC) score <3), brain haemorrhage or multiple sclerosis with a minimum of six months after the acute infarction/onset of the disease, passive extension of the ankle to at least at neutral position, free walking without any aid for at least 20 meters in less than 2 minutes, a walking speed of ≤ 1,2m/sec (measured with 10 meter walking test) and use of surface electrical stimulation for at least two months. Furthermore, patients needed to be able to stand freely. Patients were not suitable for implantation, if they had any damage to the peripheral nervous system, suffered from uncontrolled epilepsy or adiposity, had problems with substance abuse or did not have the cognitive ability to follow medical instructions. Additional exclusion criteria were pregnancy and use of other implanted devices as well as an instable ankle joint or fixed contracture. MP, OS and OCA screened the patients before inclusion. Out of ten patients who were initially included in the study, eight finished the baseline and follow-up measurements as shown in Fig 1. One patient was excluded from the assessment at the time of the baseline measurement because of pre-existing circulatory problems during standing, which made it impossible to prepare her for gait analysis. Nevertheless, she received an implant and could use it in daily life without any side effects. The second patient was excluded since she had a fall a few months after implantation, where the peroneal nerve was compressed, which led to neurapraxia. After nerve healing and a delay of one year she was able to use the system again. There was no need for revision surgery. Table 1 summarizes the demographic and clinical characteristics of all patients at the time of inclusion as well as the characteristics for the patients included in analysis. One of the remaining eight patients refused to walk without the ActiGait system at follow-up, while another one was not willing to walk without surface stimulation at baseline. Their gait analysis data were included for statistical calculations, where possible and appropriate. Therefore, while for most calculations the full number of included patients (n = 8) was used, for comparisons with the baseline gait analysis without stimulation, only 7 patients could be included in analysis.

**Table 1:** Baseline characteristics of all included patients (n = 10) and those used for analysis (n = 8).

	Gender	Age (years)	Body weight (kg)	Body height (cm)	Cause of hemiparesis	Affected side	Surface stimulation system used
**Mean ± SD n = 10**	Male: 5 Female: 5	48.6 ± 16.2	80.0 ± 19.6	174.6 ± 9.1	Haemorrhagic Stroke: 3 Ischemic Stroke:6 Multiple Sclerosis: 1	left: 4 right: 6	OS: 5 MG: 4 BN: 1
**Mean ± SD n = 8**	Male: 5 Female: 3	49.8 ± 13.4	85.6 ± 17.8	175.4 ± 9.0	Haemorrhagic Stroke: 3 Ischemic Stroke: 4 Multiple Sclerosis: 1	left: 3 right: 5	OS: 5 MG: 2 BN: 1

The systems used for surface stimulation were the Odstock 2-channel stimulator (Odstock Medical Ltd, Salisbury, UK; OS), Mygait (Ottobock Health Care, Duderstadt, Germany; MG) and Bioness L300 (Bioness Inc., Zwijndrecht, The Netherlands; BN).

### Study design

In this longitudinal clinical assessment, a participant’s gait was assessed two times with two different conditions each. Rated as gold standard for investigating changes in gait [33–36], a computerized video-based motion analysis system with infrared cameras was used to track changes in pre-defined spatiotemporal parameters. The precision of this system has been shown in various publications [37–39]. At baseline, the video-based gait analysis was performed, first with no assistive device and subsequently with the surface electrical stimulation unit for supporting dorsal extension of the foot during the swing phase of walking. Patients were asked to walk with a comfortable walking speed comparable to their daily lives. Additionally, a 10-meter walking test was performed with the fastest convenient walking speed, which has been reported to have a good responsiveness to changes in rehabilitation after stroke [40]. The Visual Analogue Scale (VAS) included in the EuroQol questionnaire (EQ-VAS) was used to record perceived health status, with 0 mm indicating the worst and 100 mm the best health condition [41]. Literature supports the validity and reliability of the EQ-VAS [42], and its applicability in a post-stroke population could already be demonstrated [43]. This quantitative measure of health perception was chosen to integrate a patient-reported outcome as well, since it is well known that objective “positive” results might not automatically translate into subjective well-being [44]. Additionally, at baseline and follow-up patients were asked within a short interview about assistive devices they used at home and if they experienced any problems with the system they were currently using. Also, patients were inspected for skin irritations in the area of stimulation by a specialist in rehabilitation medicine.

Within two months after baseline measurement, the CE-certified system ActiGait (Ottobock Health Care, Duderstadt, Germany) was surgically implanted. Surgery in all patients was performed by the senior author (OCA).

Follow-up measurements were conducted one year after implantation of the device. They included a video-based gait analysis with both the system switched on and off, as well as a 10-meter walking test, the short interview, and the VAS for health status. For the instrumented gait analysis as well as for the 10-meter walking test, patients were tested without stimulation first and with their implanted system in use afterwards. This approach was chosen to ensure the same conditions at baseline and follow-up. Additionally, it ensured that patients were not fatigued when walking without stimulation. As the patients had the possibility to walk along the gait analysis course a few times before data were collected, a learning effect within the measures was deemed unlikely. The measurement period was between July 26, 2012 to February 29, 2016.

The maximum dorsal extension of the ankle at initial contact, the cadence and the step length of the affected side were defined as the main outcome parameters. While the first-mentioned is a relevant indicator for sufficient tibialis anterior muscle function (and therefore sufficiency of the stimulation), the other two provide important detailed information on walking ability and allow to understand where potential changes in walking speed originate from.

The major muscle activation of the tibialis anterior is through the swing phase with an increase towards the end of the gait cycle and a peak activation level in the moment of the initial contactin healthy people [45, 46]. The stroke survivors included in this study could not extend their ankle joint dorsally while walking due to insufficient muscle strength/activation. However, muscle activation itself, both voluntary and stimulated, does not necessarily mean that the supposedly affected joint–here the ankle–will move sufficiently. Either joint mobility might be impaired or co-stimulation of the peroneii might lead to primarily external rotation (in case of a flawed electrode position). Therefore, the choice of peak dorsal extension angle at initial contactwas chosen as primary parameter, because it assured the functional sufficiency of the stimulation.

### Device and surgical procedure

All patients received the partly implantable device ActiGait. As described elsewhere [28, 29] the CE-certified device compromises of an implanted 4-channel stimulation cuff electrode for the peroneal nerve as well as an external control unit with an antenna transmitting signals to the implanted part. A heel switch is used to control the timing of the stimulation. Lifting the heel of the affected leg triggers stimulation, while the first heel contact after swing phase stops it. However, other settings or pre-set delays in stimulation are possible. Using the external control unit, patients can switch the system on and off or change the amplitude of stimulation.

**Fig 2:**
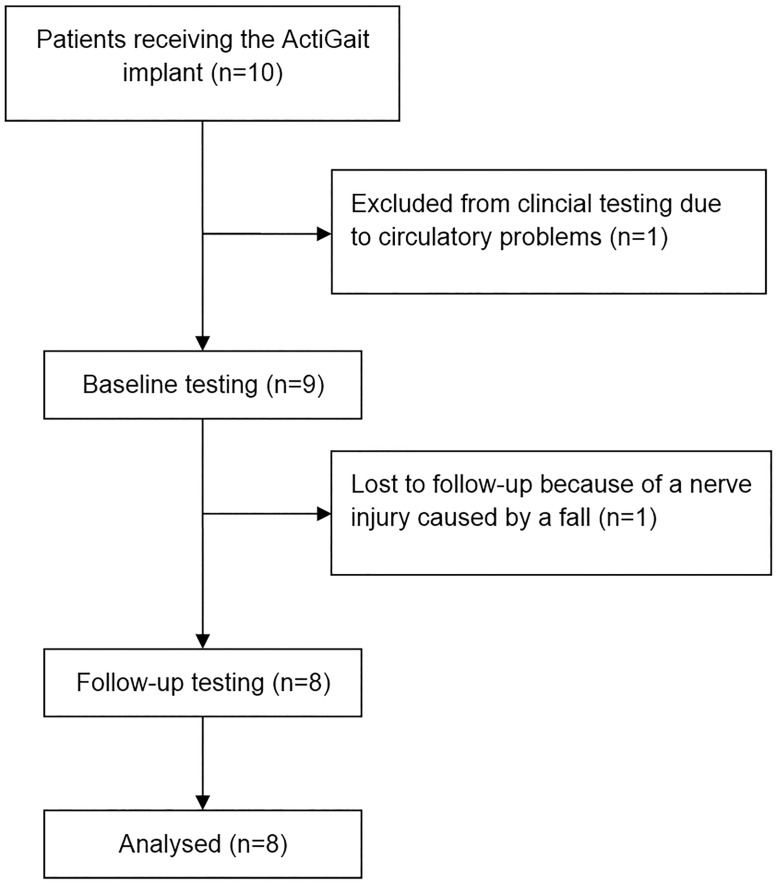
Using an incision proximal to the popliteal fossa, the cuff electrode of the ActiGait^®^ system is fixed around the motor part of the common peroneal nerve.

Implantation of the device was performed under general anaesthesia in a lateral position. Through a longitudinal incision proximal to the popliteal fossa the common peroneal nerve was exposed. Using a nerve stimulator, the sensory branches were divided under loop magnification to avoid uncomfortable sensation during stimulation of the ActiGait system. The cuff electrode was then positioned around the motor part of the common peroneal nerve as shown in Fig 2. To enable gliding of the cuff electrode a neurolysis was performed approximately 4cm proximally and distally of the electrode. The appropriate size of the cuff electrode was determined using dummies with different diameters, thus the smallest one, where gliding was still possible, was used. The stimulator body was inserted through a separate incision distally to the greater trochanter region of the femur and was sutured to the lateral femoral fascia. At the end of the operation radiographs were taken in flexed and extended knee positions to control the correct placement of the cuff and the system was tested using the external unit. The detailed implantation procedure is described elsewhere [28].

The device was activated four to five weeks after implantation. Within the first six weeks after activation we recommended to use the device for not longer than two hours a day to avoid overuse symptoms (such as tendopathies) or muscle fatigue. Afterwards, patients were asked to slowly increase the stimulation time until they were able to use the device during the whole day.

Device settings were selected individually for each patient. The stimulation frequency for all patients was chosen between 20 and 40 Hz with a current of 1.1mA. Patients could modify the stimulation intensity by regulating the pulse width during device usage. The choice of the stimulation channel(s) was determined by clinical tests for each individual. Channel one and four were used most frequently as they led to a greater dorsal extension of the foot while channel two and three usually resulted in stimulation of the peroneus muscles.

### Gait analysis

**Fig 3:**
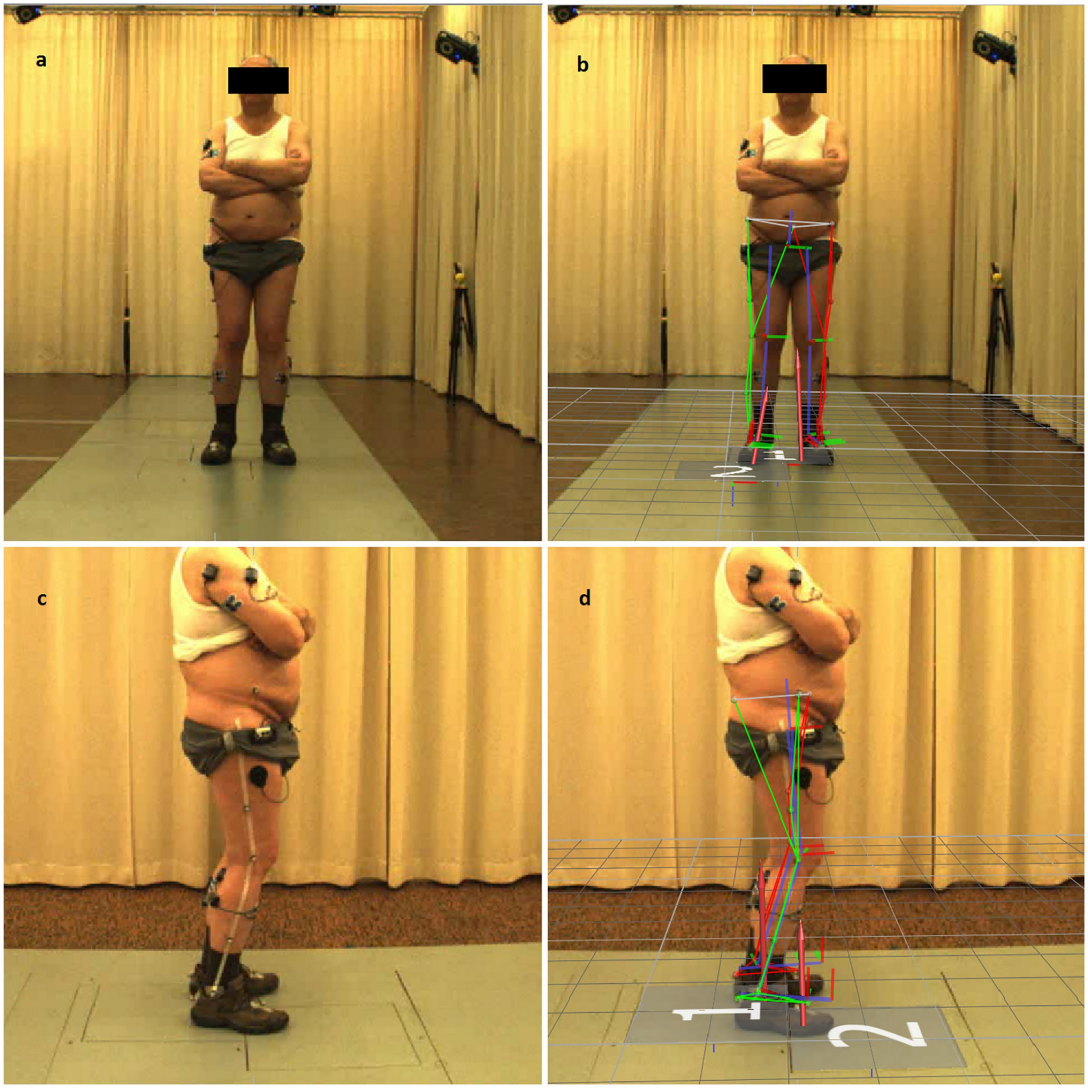
Experimental setup of the 3D motion analysis. Placement of the infrared-reflecting markers on a patient in frontal view without (a) and with (b) overlay of the model used by the software to calculate the kinematic model. At the lower half of the screen (c&d), the markers are shown from a sagittal view. The overlay (b&d) also highlights the embedded force plates (marked with 1&2) that were intended to be used for calculating kinetic data.

Gait analysis at baseline and follow-up was performed with a computerized video-based motion analysis system consisting of eight infrared cameras (Vicon Nexus 1.7.1, Motion Systems Limited, MX T10—Cameras, Oxford, UK, 200 Hz) and two high speed video cameras (Basler AG, Vision Technologies, Pilot, piA640 –210gc, Ahrensburg, Germany, 200 Hz) while participants were walking on a standard walking course of eight meters length. Kinematic data of the hip, knee and ankle were obtained using a modified Helen-Hayes marker set (Vicon Nexus, PlugIn Gait SACR, 16mm) for the lower body [46] as presented in Fig 3. Due to an insufficient number of clean hits on the two embedded force plates (AMTI, Watertown, MA, USA, 1000 Hz), kinetic data could not be reported. Surface electromyographic measurements were conducted for the tibialis anterior muscle and the gastrocnemius on both legs, but were not included in analysis as data validation showed high zero offsets and/or big variations between the gait cycles for some patients. Raw data was processed with specific gait analysis software (Vicon Nexus 1.7.1 and Polygon 4.1, Oxford, UK). For surface stimulation, the system was set up by either a specialist in Physical and Rehabilitation Medicine or a neurologist, who are both trained with surface stimulation systems. The markers and EMG electrodes necessary for 3D motion analysis were placed by a team of two physiotherapists and a sports scientist experienced with the system. To enhance validity of outcome data, the same team placed the markers/electrodes for all participants and conditions.

### Statistics

Statistical analysis was performed in SPSS 24. Data was checked for normal distribution using a Kolmogorov-Smirnov test. As only a few parameters showed normal distribution, non-parametric tests using paired measures were used to detect significant changes in the main outcome parameters. Following the Bonferroni Holm Procedure to correct for multiple testing, the p-values for the three main outcome parameters were set to 0.05, 0.025 and 0.017, respectively. In the other outcome parameters descriptive methods were applied.

## Results

### Main outcome parameters

A statistically significant change was detected in the main kinematic outcome parameter, the dorsal extension of the ankle at initial contact. It increased significantly between baseline without stimulation and follow-up with ActiGait (p = 0.018; mean -4.6° vs. 5.7°) as shown in Table 2. Additionally, analysis showed similar changes in ankle dorsal extension between walking without electrical stimulation compared to surface stimulation (p = 0.028; mean -4.6° vs. 2.1°).

**Table 2:** Main outcome parameters of the affected leg including the dorsal extension of the ankle, the steps per minute and the length of each step.

Parameter (mean ± SD)	Baseline without stimulation	Baseline with surface electrical stimulation	Follow-up without stimulation	Follow-up with active ActiGait	Comparison of baseline without stimulation and with stimulation (n = 7)	Comparison of baseline without stimulation and follow-up with ActiGait (n = 7)
**Dorsal extension of the ankle at initial contact (°)**	-4.6 ± 7.5	2.1 ± 9.6	-3.9 ± 6.2	5,7 ± 8.3	p = 0.028	p = 0.018
**Cadence (steps/min)**	82.7 ± 27.4	80.6 ± 25.4	76.1 ± 21.2	83.5 ± 19.1	p = 0.735	p = 0.31
**Step length (m)**	0.45 ± 0.18	0.45 ± 0.17	0.45 ± 0.16	0.50 ± 0.16	p = 0.553	p = 0.075

All parameters were measured at baseline and one-year follow up. P-values for the comparison between baseline without stimulation and baseline/follow up with stimulation are reported in the right-hand columns.

There was no statistically significant change in the cadence between baseline without stimulation and follow-up with ActiGait (p = 0.31; mean 82.7 steps/min vs. 83.5 steps/min) and in the step length of the affected leg (p = 0.075, mean 0.45m vs. 0.50m), as presented in Table 2.

**Fig 4:**
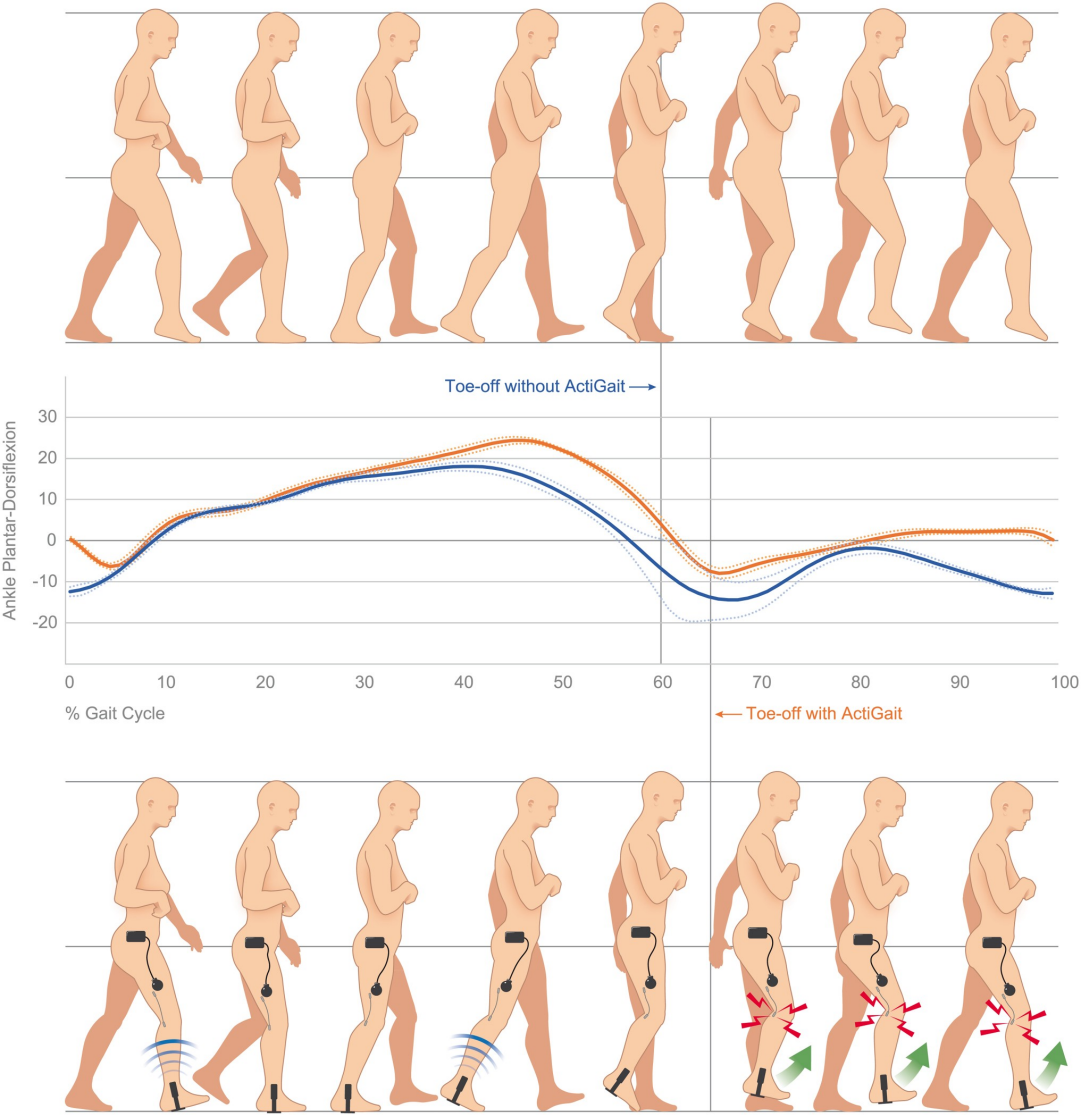
Visualization of the kinematics of the ankle joint in one patient during walking without electrical stimulation (illustration at top, blue line) at baseline and with ActiGait after one year (illustration at the bottom, orange line). The more impaired leg is presented as the right leg: Without electrical stimulation (blue line), the patient does not have the ability to actively extend the ankle at the end of swing phase, which results in a drop foot and an initial contact with toes first. Additionally, the large standard deviation after toe-off without electrical stimulation indicates an unstable movement pattern when lifting the leg. In contrast to that, the patient is able to maintain dorsal extension of the ankle during swing phase with ActiGait (orange line) and the initial contact is with the heel. This is closer to the healthy norm.

Within analysis of the ankle kinematics during the gait cycle, plantarflexion was seen in all patients at the end of swing phase without an assistive device. In contrast to that, patients showed dorsal extension or a neutral position at initial contact when ActiGait was used. Movement in the ankle joint during the gait cycle is displayed in Fig 4 for one patient without stimulation and with the ActiGait system. While this figure does only report on the changes for a single patient, it explains the fundamental functionality of the system. Additionally, it shows how stimulation might affect gait parameters during the whole gait cycle, while parameters in Table 2 give an impression of mean changes of specific gait features for all participants.

### Secondary outcome parameters

#### Kinematics

The maximum flexion of the knee during swing phase on the affected leg increased from 36.5° at baseline without stimulation to 39.8° at follow-up with ActiGait (p = 0.028). Also, we documented an increase of maximum knee flexion on the contralateral side during swing phase (54.4° vs. 61.2°, p = 0.028). During swing phase the maximum dorsal extension of the affected ankle increased from 3.0° ± 8.3° to 8.9° ± 7.1° with surface electrical stimulation (p = 0.028) and to 10.0° ± 3.0° with the implanted system (p = 0.028). There was no change in the dorsal extension of the contralateral ankle at initial contact (4.1° ± 6.6° at baseline and 7.3° ± 5.5° at follow-up with ActiGait, p = 0.310), and no change in maximal dorsal extension of the contralateral ankle (8.9° ± 4.0° at baseline and 10.2° ± 2.8°, p = 1.00). The changes in maximal flexion of the affected hip in swing phase from baseline (31.2° ± 17.1°) to surface electrical stimulation (33.7° ± 15.4°) and to the implanted system (27.5° ± 13.1°) were not significant (p = 0.236 and p = 0.352, respectively). The same was true for the maximal flexion of the less-affected hip in swing phase. These results are presented in detail in Table 3.

**Table 3:** Mean scores of patients’ kinematic gait parameters for baseline and one-year follow up.

Parameter (mean ± SD)	Baseline without stimulation	Baseline with surface electrical stimulation	Follow-up without stimulation	Follow-up with active ActiGait	Comparison of baseline without stimulation and with stimulation (n = 7)	Comparison of baseline without stimulation and follow-up with ActiGait (n = 7)
**Maximum flexion of the affected knee during swing phase (°)**	36.5 ± 13.8	41.1 ± 13.3	40.5 ± 15.4	39.8 ± 13.9	p = 0.204	p = 0.028
**Dorsal extension of the less-affected ankle at initial contact (°)**	4.1 ± 6.6	2.6 ± 5.9	7.4 ± 3.4	7.3 ± 5.5	p = 0.612	p = 0.310
**Maximum flexion of the less-affected knee during swing phase (°)**	54.4 ± 12.6	57.0 ± 10.3	59.3 ± 11.8	61.2 ± 13.2	p = 0.345	p = 0.028
**Maximal dorsal extension of the affected ankle in swing phase (°)**	3.0 ± 8.3	8.9 ± 7.1	1.5 ± 6.4	10.0 ± 3.0	p = 0.028	p = 0.028
**Maximal dorsal extension of the less-affected ankle in swing phase (°)**	8.9 ± 4.0	9.1 ± 5.0	10.4 ± 2.4	10.2 ± 2.8	p = 0.735	p = 1.00
**Maximal flexion of the affected hip in swing phase (°)**	31.2 ± 17.1	33.7 ± 15.4	26.9 ± 14.1	27.5 ± 13.1	p = 0.236	p = 0.352
**Maximal flexion of the less-affected hip in swing phase (°)**	35.0 ± 8.6	35.8 ± 7.6	31.6 ± 8.3	30.6 ± 11.0	p = 0.866	p = 0.063

The “affected side” means the side of the body, where the patients were impaired more in their ability for dorsal extension. The contralateral side is described as “less-affected” side. P-values for the comparison between baseline without stimulation and baseline/follow up with stimulation are reported in the right-hand columns. Although not used for statistical purposes, the values for walking without stimulation at follow-up are reported for data completeness.

#### Spatio-temporal parameters

In the comparison between no stimulation and stimulation (surface or ActiGait), no relevant effects were found in the spatio-temporal parameters walking speed, the limp index, step length, step time and double support. However, there was a relevant effect in the time of single support of the affected and the contralateral leg comparing no stimulation and surface stimulation. Interestingly, these effects were not seen for the ActiGait system. All analysed spatio-temporal parameters are presented in Table 4.

**Table 4:** Mean scores of patients’ spatio-temporal gait parameters.

Parameter (mean ± SD)	Baseline without stimulation	Baseline with surface electrical stimulation	Follow-up without stimulation	Follow-up with active ActiGait	Comparison of baseline without stimulation and with stimulation (n = 7)	Comparison of baseline without stimulation and follow-up with ActiGait (n = 7)
**Comfortable walking speed (m/s)**	0.67 ± 0.34	0.66 ± 0.29	0.58 ± 0.29	0.69 ± 0.28	p = 0.498	p = 0.463
**Single support of the affected leg (s)**	0.40 ± 0.08	0.45 ± 0.10	0.38 ± 0.06	0.40 ± 0.08	p = 0.046	p = 0.497
**Single support of the contralateral leg (s)**	0.59 ± 0.16	0.59 ± 0.14	0.58 ± 0.11	0.55 ± 0.09	p = 0.018	p = 0.128
**Double support (s)**	0.66 ± 0.56	0.65 ± 0.51	0.76 ± 0.42	0.59 ± 0.28	p = 0.397	p = 0.398
**Step time of the affected leg (s)**	0.92 ± 0.32	0.88 ± 0.23	0.93 ± 0.23	0.84 ± 0.19	p = 0.141	p = 0.204
**Step time of the contralateral leg (s)**	0.76 ± 0.46	0.81 ± 0.51	0.80 ± 0.34	0.68 ± 0.24	p = 0.735	p = 0.345
**Step length of the contralateral leg (m)**	0.46 ± 0.10	0.48 ± 0.07	0.41 ± 0.13	0.45 ± 0.09	p = 0.207	p = 0.671
**Limp index**	0.85 ± 0.08	0.86 ± 0.05	0.84 ± 0.07	0.88 ± 0.08	p = 0.552	p = 0.127

Within the time-space parameters “single support” means the time during the gait cycle the whole body weight relies on one leg only, while “double support” is the time both feet have ground contact. “Step time” describes the time needed to move one foot forward, while “step length” describes the covered distance while taking one step. The limp index is a measure of gait (a)symmetry with “1” indicating a perfectly symmetric gait.

#### 10-meter walking test

While comfortable walking speed was assessed within 3D gait analysis, a 10-meter walking test was used to assess changes in the fastest convenient walking speed. At baseline without stimulation participants (n = 8) needed a mean time of 20.1 ± 8.5 s for walking 10 meters at their fastest convenient walking speed. This parameter improved in both conditions, walking with surface stimulation (17.3 ± 7.5s) and walking with ActiGait (16.3 ± 6.5s), resulting in statistically significant differences between baseline without stimulation and surface stimulation (p = 0.021) as well as ActiGait (p = 0.036). These changes are also clinically meaningful for this population of patients [47].

#### Self-reported health, assistive devices and problems with device use

A comparison of the self-reported health status (EQ VAS) before implantation and at follow-up showed a significant improvement. While participants (n = 8) rated their health status on a 100 mm Visual Analogue Scale with 49 (±14) mm before intervention, their rating was 74 (±15) mm after using ActiGait for one year (p = 0.028). As shown in Table 5, two out of eight patients were found to have skin problems in the area of stimulation with surface stimulation and one reported problems with the use of the system (electrode displacement in particular). These problems could be solved with the use of an implanted system. While a majority of these patients reported to have had skin problems at any point with their surface system, this information was not included in further analysis due to the limited possibilities to verify these anectodical reports. Additionally, one stroke survivor who regularly used a walking stick at baseline did not need it anymore at 1-year follow-up.

**Table 5:** Number of stroke survivors experiencing problems with skin irritation and system as well as use of assistive devices (total n = 8).

	Current skin problems in the area of stimulation	Issues with electrode placement or use of the system	Current use of any walking aid in daily life
At baseline	2	1	3
After using ActiGait for one year	0	0	2

## Discussion

As drop foot is a frequent problem in patients with neurologic conditions [1], different assistive devices were designed to improve dorsal extension of the foot while walking [27, 48]. While previous studies could confirm good functional outcomes and patient satisfaction with implanted FES systems within relatively short follow-up periods, we were the first to investigate outcomes of such a system with a one-year follow-up. Gait parameters during surface stimulation and the use of an implanted system were compared to walking without an assistive device. While the tested system is currently not available on the market, this study evaluating its efficacy, long-term outcome and safety is relevant for further product development of implantable systems. Additionally, regardless of the application method, this study contributes to the understanding of the potential long-term effects of FES in general.

Gait analysis showed that compared to no stimulation the implanted device enabled more dorsal extension of the ankle of the affected leg during swing phase and at initial contact as charted in Fig 4, where specific changes in one patient are illustrated. These findings from the first study reporting on a 1-year follow-up are in line with previous research investigating short-term effects of the device [28, 29, 31]. The improved dorsal extension of the ankle at initial contact allowed the heel to be the first contact with the ground as described for physiological gait [2, 3]. This results in placing the ground reaction force vector posterior to the ankle which allows a first arc of passive plantarflexion after initial contact while the heel remains the main site of foot support [49]. This is important to enable weight transfer to the foot and to absorb forces during the heel strike [50, 51]. While the heel pad and ankle joint are anatomical structures that can absorb these forces if the heel is the first contact with the ground [51, 52], included patients could not use this natural protection against the transient forces without electrical stimulation as their drop foot resulted in an unstable initial contact with toes first. In contrast to that, ActiGait as well as surface stimulation allowed normal initial contact with the heel as shown in Fig 4 and Table 1. The neutral position of the ankle joint at initial contact (as indicated in Fig 4 while using the ActiGait system) is within the norm values presented in standard gait analysis literature [2, 3, 49]

An improved dorsal extension of the foot during swing in addition to improved knee flexion was recorded while using surface stimulation or the ActiGait system. This allows better clearance while walking and therefore may result in a reduced risk of fall.

While the evaluation of falls and sense of safety were not directly evaluated within this study, the use of walking aids can be seen as an indirect measure for mobility and sense of safety. Here, one of the three patients using walking aids at baseline, ceased them, which freed both hands for daily-life activities such as carrying shopping bags.

Additionally, a Visual Analogue Scale for health status (EQ-VAS) was used. Here, patients reported an improved health status (mean change 24mm) at follow-up, which may also be an indicator for a higher mobility when using the ActiGait system. A greater reported walking distance with ActiGait, was found in a case study of Hausmann et al. [30], while an improved quality of life was also reported by Burridge et al. [26] and Martin et al. [32, 53]. In this study, the improved health status might also be an indicator for satisfaction with the device. Although satisfaction was not directly measured, problems experienced with the surface stimulation system, as skin irritation and electrode displacement, could be resolved with the implanted system. As there were no problems reported with the handling of ActiGait at follow-up, we conclude that usability was better than with the surface system.

Within 3D gait analysis, we observed no significant differences in step length of the affected side when using ActiGait (p = 0.075) or surface stimulation (p = 0.553). Furthermore, we did not find a greater cadence and a relevant improvement in walking speed was seen only in the fast speed 10-meter walking test, but not during VICON gait analysis, for all subjects. This can be explained by the different instructions given for both conditions. While patients were asked to walk at a comfortable close-to-daily-life walking speed during the instrumented gait analysis, they were asked to walk at their fastest convenient and safe walking speed during the 10-meter walking test. As the cadence was already near the lower limit of an age-matched norm [54] in the comfortable walking speed condition, this might have been the most efficient walking speed. Nevertheless, a better sense of safety might have allowed them to walk faster at the 10-meter walking test, when they were instructed to do so. Most previous studies on implanted and surface electrical stimulation systems evaluated walking speed during a 10-meter walking test. However, instructions for patients varied within these studies, with some measuring the fastest possible walking speed [29, 56], others asking patients to walk at a comfortable speed [27, 31] or not specifying this in their publication [30]. Our data indicates that these instructions make a difference and should be specified to enhance validity and allow comparison between studies.

Another interesting finding of this study was that most of the changes in gait parameters seen with ActiGait could already be detected with surface electrical stimulation. This adds evidence to clinical guidelines that suggest the use of surface stimulation before considering an implanted solution. If the patient is satisfied with the device, we see no need to undergo surgery for an implanted solution. However, if there are skin problems [25] which have been reported by several patients or habitual surface electrode displacement, implanted peroneal stimulation may be considered.

The main limitation of this study was the small sample size. While it is comparable to other studies on the topic [29–31], the explanatory power and the external validity is still limited. Due to the limited number of subjects a generalisation of the results for the population of stroke survivors with gait abnormality due to drop foot is not possible as there was no testing for different age groups, gender or other factors. While on one hand, positive outliers could have led to an overestimation of effects, the presentation of false-positive results, as described as a risk factor of small sample sizes [56], this is highly unlikely, since dorsal extension of the foot increased significantly using a 95-% confidence interval and spontaneous improvements in dorsal extension could not have been the case, since testing without stimulation yielded almost identical results at baseline and after one year.

On the other hand an under-estimation of the effect of the device is more likely as smaller sample sizes require larger effect sizes to show significant results. This possible under-estimation of the effects might explain why we did not find any significant difference in cadence, while previous studies reported a significantly higher cadence while using implantable FES. In further studies a larger sample size might allow to directly test correlations of gait parameters between surface stimulation and an implanted system, which was not possible here.

## Conclusion

We investigated gait changes in subjects with a neurologic condition causing drop foot when using surface electrical stimulation or nerve stimulation with an implanted device with a follow-up period of one year. A significant improvement in dorsal extension of the ankle was seen at initial contact with both stimulation approaches, which occurred with heel first re-approaching as in healthy gait. Increased dorsal extension during swing phase allowed proper foot clearance. While this is in line with previous research with short follow-up results, this is the first study to present long-term results of an implanted nerve stimulatory device. We could conclude that the device was safe in its long-term use and showed that changes in gait parameters with implanted stimulation are similar to those with surface electrical stimulation. For patients with skin irritations and difficulties with electrode positioning in surface systems implanted systems may therefore be considered as an alternative for long-term use.

## Supporting information

S1 File Original study protocol in German. (PDF)

S2 File English translation of the original study protocol. (PDF)
