# Supplementary material for: The long-term effects of an implantable drop foot stimulator on gait in hemiparetic patients
Source: PLoS One. 2019 Apr 17;14(4):e0214991. doi: 10.1371/journal.pone.0214991 (PMC6469760; doi:10.1371/journal.pone.0214991)
Supplement: S1 File — (PDF) [file pone.0214991.s001.pdf]

Protokoll zu „Retrospektive Datenanalyse von PatientInnen mit implantierten N.peroneus-Stimulator“

Version 1.1

Erstellt am 16.12.2016

Sponsor: Medizinische Universität Wien
Christian Doppler Labor zur Wiederherstellung von Extremitätenfunktionen
Spitalgasse 23
1090 Wien

Kontaktperson: Univ.-Prof. Dr. Oskar C. Aszmann

Projektleiter: Agnes Sturma, M.Sc. B.sc.

Physiotherapeutin
im Christian Doppler Labor zur Wiederherstellung von Extremitätenfunktionen
Spitalgasse 23
1090 Wien

+43/664 9115815
agnes.sturma@meduniwien.ac.at

Prüfarzt: Univ.-Prof. Dr. Oskar C. Aszmann
Plastische und Rekonstruktive Chirurgie
Medizinische Universität Wien
Spitalgasse 23
1090 Wien

01/40 400 69940
oskar.aszmann@meduniwien.ac.at

Wegen der besseren Lesbarkeit wird im weiteren Text zum Teil auf die gleichzeitige Verwendung weiblicher und männlicher Personenbegriffe verzichtet. Gemeint und angesprochen sind – sofern zutreffend – immer beide Geschlechter.

Einführung:

Das Christian Doppler Labor zur Wiederherstellung von Extremitätenfunktion an der Medizinischen Universität Wien wurde 2012 gegründet und befasst sich seitdem klinisch wie wissenschaftlich mit therapeutischen Möglichkeiten bei Extremitätenverlust oder –funktionsverlust.

Neben vielen anderen motorischen Einschränkungen zeigt sich bei Patienten nach Schlaganfall oft ein sogenannter „Fallfuß". Aufgrund einer Schwäche der Sprunggelenksextensoren kann dabei in der Schwungbeinphase der Vorfuß nicht ausreichend angehoben werden. Dies führt zu Ausweichbewegungen in Knie, Hüfte und Rumpf, einer verkürzten Schrittlänge, sowie einer erhöhten Sturzgefahr. Um dem entgegenzuwirken ist funktionelle Elektrostimulation des N. peroneus seit vielen Jahren in der klinischen Behandlung etabliert. Neben der Stimulation über die Hautoberfläche [1 2] gibt es seit einigen Jahren auch die Möglichkeit die Muskeln mittels implantierter Elektrostimulatoren den N. peroneus direkt zu aktivieren. Während der funktionelle Gewinn mit beiden Systemarten vergleichbar ist, ermöglicht die Implantation eine bessere Einstellung und leichtere Handhabung der Stimulation. [3] Außerdem kommt es zu keiner Reizung der Haut, die eine mögliche Nebenwirkung von transkutaner Elektrostimulation ist. Aus diesen Gründen wurden im AKH/Meduni Wien seit 2012 einige Systeme für direkte N.peroneus-Stimulation bei Patienten mit neurologischer Problematik und Fallfuß implantiert. Dabei wurde auf das CE-zertifizierte Medizinprodukt ActiGait (Neurodan, Dänemark, Otto Bock Group, 2006) zurückgegriffen.

Zielsetzung:

Um den klinischen Outcome der beschriebenen Patientengruppe evaluieren zu können und den Verbesserungsprozess zu dokumentieren, wurden im klinischen Setting in Kooperation mit der Klinik für Physikalischen Medizin und Rehabilitation bisher bereits Untersuchungen der Nervenleitgeschwindigkeit und der Gehfähigkeit durchgeführt. Diese prä- und postoperativ erhobenen Daten sollen nun im Zuge einer retrospektiven Datenanalyse anonymisiert aufgearbeitet und publiziert werden. Im Gegensatz zu einem Großteil der publizierten Literatur zu diesem Thema stehen hier Daten bzgl. der Langzeitwirkung (letzter Follow-Up ein Jahr postoperativ) zur Verfügung. [3-6] Da das Ziel der Implantation eine Langzeit-Anwendung ist, sind diese Daten für die Evaluierung äußerst wertvoll.

Verwendete Tests:

Nervenleitgeschwindigkeit (NLG):
Mittels NLG kann eine Aussage über den Zustand peripherer Nerven getroffen werden. Da ein intakter Nerv eine notwendige Voraussetzung für das Funktionieren des Systems ist, wurde präoperativ gemessen, ob eine ausreichende Funktion des N.peroneus vorhanden ist. Nach der Implantation erfolgten im Verlauf weitere NLG-Messungen um einen Nervenschaden durch Operation oder Implantat ausschließen zu können.

Instrumentelle Ganganalyse mit VICON-System:
Die Ganganalyse mittels eines VICONS-Systems ermöglicht eine genaue Analyse der Gangparameter und Gelenksstellungen während des Gehens. So kann im Detail das Gangbild von Probanden beschreiben werden. Dazu werden an genau definierten anatomischen Punkten des Probanden (z.B.

Spina iliaca anterior superior, Malleolus lateralis,…) an der Hautoberfläche Infrarot-reflektierende Marker angebracht. Diese werden von Infrarot-Kameras im Zeitverlauf erfasst. Anschließend kann software-unterstützt berechnet werden, wie die einzelnen Körpersegmente während des Gangzyklus zueinanderstehen (Kinematik) und mit welcher Geschwindigkeit, Schrittanzahl etc. (spatio-temporale Daten) sich der Proband bewegt hat. Der zusätzliche Einsatz von Oberflächen-EMG-Ableitungen bietet außerdem noch die Möglichkeit zusätzlich eine Aussage über die Muskelaktivierung während des Ganges zu treffen.

<u>10 m-Walking Test:</u>
Der 10-Meter-Geh-Test ist ein gut validierter und klinisch einfach durchführbarer Test um einen Grobüberblick über die Gehfähigkeit von Personen zu bekommen. Während der Proband eine Strecke von 10m geht, wird dabei die Zeit gestoppt. So kann schnell erhoben werden, ob die Geh-Geschwindigkeit im Normbereich liegt.

Teilnehmer:

<u>Einschlusskriterien:</u> In die Datenanalyse werden alle verfügbaren 10 Datensätze eingeschlossen. Patienten wurden für die ActiGait-Implantation vorgesehen, wenn sie aufgrund ihrer neurologischen Problematik einen Fallfuß hatten und das Akutereignis mindestens 6 Monate zurücklag. Voraussetzung war zusätzlich eine aktive Gehfähigkeit, eine gute passive Beweglichkeit des Sprunggelenks und eine Nervenleitgeschwindigkeit im Normbereich. Die Gehgeschwindigkeit durfte nicht größer als 1,2m/sek sein. Außerdem wurden nur Patienten eingeschlossen, die durch transkutane funktionelle Elektrostimulation eine Verbesserung des Gangbildes hatten und diese bereits zumindest 2 Monate lang regelmäßig angewendet haben.

<u>Ausschlusskriterien:</u> Von der Implantation ausgeschlossen sind Personen mit einer zusätzlichen Verletzung/Erkrankung des N. peroneus, Herzschrittmacher oder anderen elektronischen Implantaten, oder mit unbehandelten psychiatrischen Erkrankungen. Weitere Ausschlusskriterien sind ein instabiles Sprunggelenk in der Standbeinphase, fixierte Kontrakturen, Diabetes mellitus (PNP) bei pathologischen NLG des N. peronaeus, chronische Nierenerkrankungen bei pathologischen NLG des N. peronaeus, periphere arterielle Verschlusskrankheit mit manifester Claudicatio 2B nach Fontaine, Adipositas, schlecht kontrollierte Epilepsie, Schwangerschaft, Alkohol- und/oder Drogenabusus, sowie deutliche kognitive Einschränkungen.

<u>Patienten:</u> Die Daten von allen 10 Patienten, die seit 2012 mit ActiGait versorgt worden sind und bei denen die beschriebenen Tests durchgeführt wurden, sollen retrospektiv analysiert werden. Im Detail handelt es sich dabei um den Messzeitraum von 26.7.2012 bis 29.2.2016. Für die Patienten kommt es zu keinen zusätzlichen Untersuchungen.

<u>Datenschutz:</u> Die aufgenommenen Daten werden anonymisiert und unter einer ID in einem Passwort-gesichertem PC gespeichert; nur offizielle Prüfer des Projektes haben auf die aufgenommen Daten Zugriff.

<u>Risiko und Nutzen:</u> Da es sich bei der Datenanalyse lediglich um die retrospektive Auswertung von bereits vorhandenen klinischen Daten handelt, gibt es durch die Studie keine gesundheitlichen Risiken für die Patienten. Das einzige Risiko bei der retrospektiven Datenanalyse ist das Bekanntwerden von personenbezogenen Gesundheitsdaten, dem mit einer Anonymisierung der Daten entgegengewirkt werden kann. Durch die Datenanalyse entsteht für die Teilnehmer per se kein Nutzen.

<u>Versicherung:</u> Da kein Risiko für die Teilnehmer besteht, ist keine Versicherung notwendig.

Entgelt: Da die Teilnehmer keinen Aufwand durch die Datenanalyse haben, ist kein Entgelt vorgesehen.

Vorgehensweise:

Jene Datensätze, die von der Universitätsklinik für Physikalische Medizin und Rehabilitation in Kooperation mit dem Christian Doppler Labor für Extremitätenfunktionen von 26.7.2012 bis 29.2.2016 von 10 Patienten erhoben wurden, sollen retrospektiv ausgewertet werden. Diese wurden jeweils präoperativ, sowie 6 und 12 Monate nach der Implantation Daten zur NLG-Messung, 10m Walking Test und instrumenteller Ganganalyse mittels VICON System erhoben.

Statistische Auswertung der erhobenen Daten:

Nullhypothese: Durch die Implantation und Nutzung von ActiGait ändert sich die Gehfähigkeit von PatientInnen mit Fallfuß nicht.

Alternativhypothese: Durch die Implantation und Nutzung von ActiGait kommt es zur Vergrößerung von Schrittlänge und Kadenz.

Es sollen parametrische und nicht-parametrische Tests eingesetzt werden um Prae-Post-Vergleiche der Gruppe anstellen zu können und Korrelation zwischen prä- und postoperativer Gehfähigkeit berechnen zu können. Als Hauptzielgröße werden Schrittlänge und Kadenz angenommen.

Da es sich um eine retrospektive Analyse klinischer Daten handelt und die Patientenanzahl nicht beeinflusst werden kann, ist eine Fallzahlberechnung nicht sinnvoll. Die angedachte Fallzahl ist aber vergleichbar mit bisher publizierten Studien zu diesem Thema, die Patienten über einen Zeitraum von jedenfalls 3 Monaten nach Implantation analysiert haben. [4-6]

In der statistischen Auswertung sollen in Einklang mit der vorhandenen Literatur [3-5] Signifikanzen mit einem Alpha-Fehler von 0.05 angegeben werden.

Im Falle einer Normalverteilung der Daten ist ein prä-post Vergleich mittels gepaartem-t-test geplant. Um eine erwartete Korrelationen zwischen der Gehfähigkeit vor der Implantation und mit ActiGait feststellen zu können wird unter der Annahme von normalverteilten Daten mit linearem Zusammenhang der Pearson-Korrelationskoeffizient ermittelt. Sollte sich die Annahme des linearen Zusammenhanges nicht bestätigen, wird eine Spearman-Korrelation der Ränge gerechnet.

Verwendung der Daten:

Es ist geplant, die erhobenen Daten anonymisiert für Publikationen in peer-reviewten Journalen oder bei Konferenzen zu verwenden.

Literatur:

1. Stein RB, Everaert DG, Thompson AK, et al. Long-term therapeutic and orthotic effects of a foot drop stimulator on walking performance in progressive and nonprogressive neurological disorders. Neurorehabil Neural Repair 2010;**24**(2):152-67 doi: 10.1177/1545968309347681[published Online First: Epub Date]|.
2. Kottink AI, Oostendorp LJ, Buurke JH, Nene AV, Hermens HJ, MJ IJ. The orthotic effect of functional electrical stimulation on the improvement of walking in stroke patients with a dropped foot: a systematic review. Artificial organs 2004;**28**(6):577-86 doi: 10.1111/j.1525-1594.2004.07310.x[published Online First: Epub Date]|.
3. Martin KD, Polanski WH, Schulz AK, et al. Restoration of ankle movements with the ActiGait implantable drop foot stimulator: a safe and reliable treatment option for permanent central leg palsy. Journal of neurosurgery 2016;**124**(1):70-6 doi: 10.3171/2014.12.jns142110[published Online First: Epub Date]|.
4. Ernst J, Grundey J, Hewitt M, et al. Towards physiological ankle movements with the ActiGait implantable drop foot stimulator in chronic stroke. Restorative neurology and neuroscience 2013;**31**(5):557-69 doi: 10.3233/rnn-120283[published Online First: Epub Date]|.
5. Schiemanck S, Berenpas F, van Swigchem R, et al. Effects of implantable peroneal nerve stimulation on gait quality, energy expenditure, participation and user satisfaction in patients with post-stroke drop foot using an ankle-foot orthosis. Restorative neurology and neuroscience 2015;**33**(6):795-807 doi: 10.3233/rnn-150501[published Online First: Epub Date]|.
6. Hausmann J, Sweeney-Reed CM, Sobieray U, et al. Functional electrical stimulation through direct 4-channel nerve stimulation to improve gait in multiple sclerosis: a feasibility study. J Neuroeng Rehabil 2015;**12**:100 doi: 10.1186/s12984-015-0096-3[published Online First: Epub Date]|.
