# Supplementary material for: The long-term effects of an implantable drop foot stimulator on gait in hemiparetic patients
Source: PLoS One. 2019 Apr 17;14(4):e0214991. doi: 10.1371/journal.pone.0214991 (PMC6469760; doi:10.1371/journal.pone.0214991)
Supplement: S2 File — (PDF) [file pone.0214991.s002.pdf]

Protocol for „Retrospective data analysis of patients with an implanted stimulator of the peroneal nerve"

Version 1.1

From December 16, 2016

[translated from German in March 2018]

Sponsor: Medical University of Vienna
Christian Doppler Laboratory for Restoration of Extremity Function
Spitalgasse 23
1090 Wien

Contact person: Univ.-Prof. Dr. Oskar C. Aszmann

Project leader: Agnes Sturma, M.Sc. B.sc.

Physiotherapist
at the Christian Doppler Laboratory for Restoration of Extremity Function
Spitalgasse 23
1090 Wien

+43/664 9115815
agnes.sturma@meduniwien.ac.at

Principal investigator (MD):

Univ.-Prof. Dr. Oskar C. Aszmann
Plastic and Reconstructive Surgery
Medical University of Vienna
Spitalgasse 23
1090 Wien

01/40 400 69940
oskar.aszmann@meduniwien.ac.at

To improve the readability of the text, only the male form will be used. If not otherwise stated, both sexes are included. [This is more relevant for the original text, as the German language has different words for female and male groups of subjects, patients, etc.]

Introduction:

The Christian Doppler Laboratory for Restoration of Extremity Function at the Medical University of Vienna was founded in 2012. Its scope is to clinically and scientifically explore therapeutic options after loss of extremities or their function.

Apart from many other impairments, many patients who suffered from stroke have a so-called "drop foot", meaning the forefoot cannot be dorsiflexed during swing phase in gait due to weakness of the extensor muscles. This leads to compensatory movements in knee, hip and trunk, a reduced step length and a higher risk of fall. Electrical stimulation of the peroneal nerve is well-established for treating this condition. Apart from surface electrical stimulation [1 2], the possibility of direct stimulation of the peroneal nerve using implanted electrodes was also established in recent years. While the functional gain for both methods is comparable, the implanted systems allow a more precise adjustment and are easier to use. [3] There is also no skin irritation, which is a possible side-effect of surface electrical stimulation. For these reasons, the Medical University of Vienna / General Hospital of Vienna have implanted some systems for direct stimulation of the peroneal nerve since 2012. They were used to treat patients with neurologic conditions and a drop foot. For implantation the CE-certified medical device ActiGait (Neurodan, Denmark, Otto Bock Group, 2006) was used.

Aim:

To be able to document the clinical outcome of these patients, examinations of nerve conduction velocity and gait where carried out in a cooperation with the clinic for physical medicine and rehabilitation. Data was collected pre- and postoperatively and is now planned to be analyzed and published within a retrospective data analysis. In contrast to most published literature, the data include long-term follow ups (last follow-up after one year). [3-6] As the aim of implantation is the long-term use of the system, this data set is valuable for evaluation.

Used assessments:

<u>Nerve conduction velocity:</u>
Measuring nerve conduction velocity allows us to describe the condition of a peripheral nerve. As an intact nerve is a requirement for functioning of the system, this method was used preoperatively to make sure that there was adequate function of the peroneal nerve. After implantation measurements of nerve conduction velocity ruled out any nerve damage through surgery or implant.

<u>Instrumental Gait Analysis with a VICON System:</u>
Gait analysis using a VICON system allows a precise analysis of gait parameters and joint angles during gait to describe the gait of subjects. Hereby, infrared light reelecting markers are mounted on defined spots on the skin (e.g. Spina iliaca anterior superior, Malleolus lateralis,…). These markers are captured by infrared cameras over time. Subsequent software-based analysis gives detailed information on the angle of different body segments during the gait cycle (kinematics) and information on gait speed, number of steps etc. (spatio-temporal data). Additional use of surface EMG further allows to detect muscle activity during gait.

10 m Walking Test:
The 10-meter walking test is a validated and feasible test to get a rough summary of a person's walking capacity. Time is taken while the subject walks a distance of 10 meters, thereby testing whether gait speed is in normal range.

Subjects:

Inclusion criteria: All available ten data sets are included in analysis. Patients were included for implantation of ActiGait if they had a drop foot caused by a neurologic condition at least six months ago. Other inclusion criteria were the active ability to walk, good passive range of motion of the ankle and a nerve conduction velocity in normal range. Walking speed was not faster than 1.2m/sec. Additionally, patients were only included if they had been using transcutaneous electrical stimulation for at least two months and saw a functional benefit from it.

Exclusion criteria: Patients are excluded from implantation if they have any additional damage/condition of the peroneal nerve, cardiac pacemaker or other electric implants, or untreated psychiatric diseases. Further exclusion criteria are an instable ankle at stance phase, fixed contractures, Diabetes or chronic renal disease in combination with reduced nerve conduction velocity, Peripheral vascular disease (PVD) with clinically manifest Claudication 2B (according to Fontaine classification), adiposity, badly or uncontrolled epilepsy, pregnancy, alcohol of drug abuse, as well as significant cognitive limitations.

Patients: Data of all ten patients, who received the ActiGait implant since 2012 and who were tested by the described assessments are planned to be retrospectively analyzed. In detail, this included the measurement period between July 26, 2012 to February 29, 2016. There will be no additional examinations for the patients.

Data protection: All recorded data will be saved on a computer with password protection in anonymized form; only official investigators of the project have access to the data.

Risks and benefits: As this is only retrospective analysis of already available clinical data, there will be no risks for the patients. The only risk of this analysis is that personal health data is made public, which can be avoided by anonymization. The data analysis itself poses no risk for the participants.

Insurance: As there is no risk for the participants, no insurance is required.

Payment: As there is no additional expenditure for the participants, no payment is planned.

Procedure:

A retrospective data analysis is planned. It will include the data sets of ten patients that were collected by the Clinic for Physical Medicine and Rehabilitation in cooperation with the Christian Doppler Laboratory for Restoration of Extremity Function between July 26, 2012 and February 29, 2016. Data was collected pre-operatively, as well as six and twelve months after implantation of the device, including data of nerve conduction velocity, 10-meter walking test and instrumented gait analysis.

Statistical analysis of the data:

H0: The implantation and use of the ActiGait system does not change the walking ability of patients with drop foot.

H1: The implantation and use of the ActiGait system leads to an increase in step length and cadence.

It is planned to use parametric and non-parametric methods to compare pre- and postoperative data within the group and to correlate walking ability pre- and postoperatively. Main outcome parameters are step length and cadence.

As this is a retrospective analysis of clinical data, the number of subjects cannot be changed and there is no sense in sample size calculations. The planned sample size, however, is similar to other published studies testing patients over a period of at least three months after implantation [4-6].

For statistics we will use a significance level of 0.05 in accordance with current literature [3-5].

If there is a normal distribution of data, a pre-post comparison is planned with a paired t-test. To examine an expected correlation between walking ability before and after implantation of the ActiGait system a Pearson correlation coefficient will be calculated, if data is normally distributed. If there is no normal distribution, a Spearman correlation will be used.

Use of data:

It is planned to use the collected data in an anonymized form for publications in peer-reviewed journals and conferences.

References:

1. Stein RB, Everaert DG, Thompson AK, et al. Long-term therapeutic and orthotic effects of a foot drop stimulator on walking performance in progressive and nonprogressive neurological disorders. Neurorehabil Neural Repair 2010;**24**(2):152-67 doi: 10.1177/1545968309347681[published Online First: Epub Date]|.
2. Kottink AI, Oostendorp LJ, Buurke JH, Nene AV, Hermens HJ, MJ IJ. The orthotic effect of functional electrical stimulation on the improvement of walking in stroke patients with a dropped foot: a systematic review. Artificial organs 2004;**28**(6):577-86 doi: 10.1111/j.1525-1594.2004.07310.x[published Online First: Epub Date]|.
3. Martin KD, Polanski WH, Schulz AK, et al. Restoration of ankle movements with the ActiGait implantable drop foot stimulator: a safe and reliable treatment option for permanent central leg palsy. Journal of neurosurgery 2016;**124**(1):70-6 doi: 10.3171/2014.12.jns142110[published Online First: Epub Date]|.
4. Ernst J, Grundey J, Hewitt M, et al. Towards physiological ankle movements with the ActiGait implantable drop foot stimulator in chronic stroke. Restorative neurology and neuroscience 2013;**31**(5):557-69 doi: 10.3233/rnn-120283[published Online First: Epub Date]|.
5. Schiemanck S, Berenpas F, van Swigchem R, et al. Effects of implantable peroneal nerve stimulation on gait quality, energy expenditure, participation and user satisfaction in patients with post-stroke drop foot using an ankle-foot orthosis. Restorative neurology and neuroscience 2015;**33**(6):795-807 doi: 10.3233/rnn-150501[published Online First: Epub Date]|.
6. Hausmann J, Sweeney-Reed CM, Sobieray U, et al. Functional electrical stimulation through direct 4-channel nerve stimulation to improve gait in multiple sclerosis: a feasibility study. J Neuroeng Rehabil 2015;**12**:100 doi: 10.1186/s12984-015-0096-3[published Online First: Epub Date]|.
